# Postural Orthostatic Tachycardia With Chronic Fatigue After HPV Vaccination as Part of the “Autoimmune/Auto-inflammatory Syndrome Induced by Adjuvants”: Case Report and Literature Review

**DOI:** 10.1177/2324709614527812

**Published:** 2014-03-18

**Authors:** Lucija Tomljenovic, Serena Colafrancesco, Carlo Perricone, Yehuda Shoenfeld

**Affiliations:** 1 Sheba Medical Center, Tel-Hashomer, Israel; 2 University of British Columbia, Vancouver, British Columbia, Canada; 3 Sapienza University of Rome, Rome, Italy; 4 Tel Aviv University, Tel Aviv, Israel

**Keywords:** Postural orthostatic tachycardia, chronic fatigue, HPV vaccine, Gardasil, ASIA syndrome, vaccine adjuvants, autoimmunity, autoantibodies

## Abstract

We report the case of a 14-year-old girl who developed postural orthostatic tachycardia syndrome (POTS) with chronic fatigue 2 months following Gardasil vaccination. The patient suffered from persistent headaches, dizziness, recurrent syncope, poor motor coordination, weakness, fatigue, myalgias, numbness, tachycardia, dyspnea, visual disturbances, phonophobia, cognitive impairment, insomnia, gastrointestinal disturbances, and a weight loss of 20 pounds. The psychiatric evaluation ruled out the possibility that her symptoms were psychogenic or related to anxiety disorders. Furthermore, the patient tested positive for ANA (1:1280), lupus anticoagulant, and antiphospholipid. On clinical examination she presented livedo reticularis and was diagnosed with Raynaud’s syndrome. This case fulfills the criteria for the autoimmune/auto-inflammatory syndrome induced by adjuvants (ASIA). Because human papillomavirus vaccination is universally recommended to teenagers and because POTS frequently results in long-term disabilities (as was the case in our patient), a thorough follow-up of patients who present with relevant complaints after vaccination is strongly recommended.

## Introduction

Postural orthostatic tachycardia syndrome (POTS) is a heterogeneous disorder of the autonomic nervous system in which a change from the supine position to an upright position causes an abnormally large increase in heart rate or tachycardia (30 bpm within 10 minutes of standing or head-up tilt).^1^ The tachycardic response in POTS is frequently accompanied by a decrease in blood flow to the brain and hence a spectrum of symptoms associated with cerebral hypoperfusion (Table 1).^1-3^ Due to the wide heterogeneity of symptoms and its frequent co-occurrence with other systemic autoimmune diseases, POTS is difficult to diagnose. Moreover, because many of POTS-related symptoms are also observed in chronic anxiety and panic disorders, POTS is frequently underdiagnosed and misdiagnosed.^2^

**Table 1.** Symptoms Associated With Postural Orthostatic Tachycardia Syndrome (POTS).^2,3^

Symptom Category	Present in Current Case
Orthostatic symptoms associated with general hypoperfusion	
Light headedness/dizziness	+
Presyncope and syncope	+
Palpitations	+
Exacerbation by exercise/exercise intolerance	+
Sense of weakness	+
Tremulousness	−
Dyspnea	+
Ventricular fibrillation	−
Myocardial infarction	−
Cold extremities	+
Chest pain	−
Exacerbation associated with menses	Not reported
Hyperhidrosis	Not reported
Loss of sweating	Not reported
Tinnitus	−
Visual disturbances	+
Nonorthostatic symptoms	
Nausea	+
Bloating	−
Diarrhea	−
Constipation	−
Abdominal pain	+
Bladder symptoms	−
Other associated symptoms	
Fatigue	+
Sleep disturbances	+
Migraines	+
Neuropathic type pain	+
Cognitive disturbances	+
Flu-like symptoms	+

POTS predominantly affects women of the childbearing age with a 5:1 female–male ratio.^2^ The estimated prevalence of POTS is at least 170/100 000. This estimate was based on the finding that 40% of patients with chronic fatigue syndrome (CFS) also suffer from POTS.^4^ Indeed, CFS is a frequent and major comorbidity in POTS.^5,6^ The 2 conditions frequently appear together, and research shows that there is a clinically identifiable subgroup of patients with CFS and orthostatic intolerance that differs from control subjects and from those with CFS without orthostatic intolerance.^4^ In agreement with these observations, Okamoto et al^7^ recently found that the majority of patients with POTS also fulfilled the criteria for CFS and that severe fatigue and CFS-defining symptoms were also common in POTS patients who did not meet all the criteria for CFS. Such typical CFS symptoms that are overrepresented in POTS patients include migraine, incapacitating fatigue, fibromyalgia, unrefreshing sleep, and impaired memory or concentration. Flu-like symptoms associated with CFS such as joint pains, tender lymph nodes, and sore throat are also present in POTS albeit with lesser prevalence.^7^ These and other similar observations indicate that POTS with CFS is not a separate clinical entity entirely distinct from POTS but rather a more severe form of this condition.^7,8^ Much like POTS, CFS affects predominantly women and can be severely disabling, profoundly impairing patients’ ability to function on a daily basis.^6,9^

Genetic as well as nongenetic factors such as trauma, bacterial or viral infection, and pregnancy may predispose to POTS.^1^ In addition, it is becoming increasingly recognized that POTS and CFS can also be triggered by various medications (ie, antihypertensive drugs, antipsychotics)^1^ and vaccines.^10-15^ Herein we describe a case of a 14-year-old girl who presented with POTS/CFS of an autoimmune origin approximately 2 months after receiving her second injection of the quadrivalent human papillomavirus (qHPV) vaccine Gardasil.

## Case Report

A 14-year-old previously healthy girl presented with flu-like symptoms, sore throat, low-grade fever, fatigue, swollen glands, and intense headaches in February 2009, approximately 2 months after her second qHPV vaccine injection. Over the course of 1 week, the headache intensified and the patient further presented with photophobia, phonophobia, altered sense of taste, diminished appetite, gait disturbances, leg weakness, and inability to walk without assistance. By March 2009, her condition worsened and she quit regular school attendance due to progressively disabling symptoms. At that time she developed syncope and incapacitating chronic fatigue. Although the patient subsequently resumed attending school (by the end of 2009), her attendance was limited to 2 hours per day due to fatigue, diminished ability to focus, weakness, and severely impaired balance and coordination. She attended school in a wheel-chair and was exhausted after the 2-hour period. Her illness continue to progress, and by the end of 2010, she had the following symptoms: persistent incapacitating headaches, dizziness, recurrent syncope, lower extremity weakness, poor motor coordination, fatigue, neck pain, joint pains, numbness in the legs, blurred vision, photophobia, phonophobia, cognitive impairment, insomnia, tachycardia, dyspnea, impaired thermoregulation, cold extremities, blush discoloration of toes, excessive hair loss, gastrointestinal (GI) disturbances, altered sense of taste, diminished appetite, and weight loss (20 pounds within 3 months of symptoms onset). The psychiatric evaluation in September 2009 ruled out the possibility that the patient’s symptom were of psychosomatic origin, and the subsequent evaluation in 2010 found no evident signs of panic and anxiety disorders.

Serological evaluations revealed a number of abnormalities, including an elevated ANA at 1:1280, a positive lupus anticoagulant, and a weakly positive antiphospholipid of 7.3 in October 2009. On clinical examination, the patient presented livedo reticularis. She was then diagnosed with an undifferentiated connective tissue disease and Raynaud’s syndrome. Serology results for Epstein–Barr virus, Lyme, Babesia, and Ehrlichia were negative. Titers to *Streptococcus pneumoniae* indicated previous exposure but were however within a normal range, thus ruling out recent exposure.

Over the course of her illness, the patient experienced a complete loss of consciousness with syncope approximately 12 times. These problems were never present prior to the onset of the illness in February 2009. On further testing, the patient was diagnosed with orthostatic intolerance. In particular, on the standing test the patient’s lowest heart rate supine was 47 bpm with a blood pressure 103/56 mm Hg. On standing, the patient’s heart rate increased immediately to 82 bpm and continued to increase to a maximum of 98 bpm after 9 minutes. According to the electrophysiologist, the patient’s recurrent syncope was thus consistent with neurally mediated hypotension, and in December 2009, she was finally diagnosed with vasovagal syncope and associated postural orthostatic tachycardia syndrome. In addition, her illness met the criteria for CSF given her persisting fatigue of over 6 months, new-onset disabling headaches, postexertional worsening of the fatigue, myalgias, cognitive dysfunction, and unrefreshing sleep (Table 1). The patient’s relevant medical history includes a family history of Raynaud’s (patient’s mother) and a personal history of headaches, dizziness, photophobia, and phonophobia in 2007, all of which however resolved completely in the same year.

## Discussion

### Autoimmune Origin of POTS and CFS

Herein we described a case that clearly fulfilled the criteria for POTS/CFS (Table 1) secondary to qHPV vaccine booster injection. An autoimmune mechanism has been suggested as a causal mechanism in both POTS and CFS due to frequent findings of autoantibodies (including ANA) in POTS/CFS patients.^16,17^ Other reported abnormalities in CFS also point to an underlying autoimmune mechanism (ie, increased levels of pro-inflammatory cytokines interleukin-1, tumor necrosis factor-α, and increased levels of nuclear factor-κB).^18^ It is estimated that up to 60% of CFS patients suffer from autoimmune responses^18^ and that both POTS and CFS frequently co-occur with systemic autoimmune disorders including multiple sclerosis,^19^ Sjorgen’s syndrome,^20^ lupus,^1,21^ and Raynaud’s.^22^ Similarly, our case was diagnosed with Raynaud’s, CFS, and neurally mediated hypotension or more specifically, POTS.

Our patient’s symptoms began manifesting approximately 2 months following vaccination. An interval of 6 weeks between exposure and outcome is often used as evidence of a plausible causal association; however, immune and autoimmune diseases are chronic diseases that more often than not have a long incubation time.^23^ For example, it was reported by Arbuckle et al that systemic lupus erythematosus (SLE) evolves slowly and progressively over many years and only when enough autoantibodies are present.^24^ In particular, autoantibodies were found in 88% of SLE patients up to 9.4 years before the clinical diagnosis of the syndrome (mean = 3.3 years).^24^ Thus, long-term persistence of elevated titers of autoantibodies was necessary for the emergence of clinically overt signs and symptoms for the diagnosis of SLE. Notably, the accumulation of autoantibodies occurred while patients were still asymptomatic.

Similarly, postvaccination adverse immune phenomena can have long latency periods (ie, month to years following immunization).^25-27^ As early as 1982, compelling evidence from epidemiological, clinical, and animal research has emerged to show that autoimmune neuropathies can occur 4 to 10 months following vaccination.^28^ In such cases the disease would first manifest with vague symptoms (ie, arthralgia, myalgia, paraesthesia, weakness—note also that these are typical ASIA symptoms; Table 2), which were frequently deemed as insignificant and thus ignored. These symptoms, otherwise known as “bridging symptoms” and consistent with a mild subclinical disease, would progress slowly and insidiously until exposure to a secondary immune stimulus. The latter would then trigger the rapid and acute clinical manifestation of the disease.^28^ In other words, it was the secondary anamnestic response that would bring about the acute overt manifestation of an already present subclinical long-term persisting disease.

**Table 2.** The Suggested Criteria of ASIA^29,30^ in the Current Case of Post-HPV Vaccine POTS/CFS.

Major Criteria	Present in Current Case
1. Exposure to an external stimuli (infection, vaccine, and/or immune adjuvants) prior to clinical manifestations	+
2. The appearance of “typical” clinical manifestations	
Myalgia, muscle weakness	+
Arthralgia and/joint pain	+
Chronic fatigue, unrefreshing sleep or sleep disturbances	+
Neurological manifestations	+
Cognitive impairment, memory loss	+
Pyrexia	−
3. Removal of inciting agent induces improvement	NA
4. Typical biopsy of involved organs	Not assessed
Minor Criteria	Present in Current Case
1. The appearance of autoantibodies	+
2. Other clinical manifestations (gastrointestinal disturbances, livedo reticularis)	+
3. Specific HLA (eg, HLA DRB1, HLA DQB1)	Not assessed
4. Evolvement of an autoimmune disease (undifferentiated connective tissue disease/Raynaud’s, probable secondary antiphospholipid syndrome)	+

Consistent with these observations, we recently described several cases of autoimmunity (systemic lupus) following Gardasil where the nonspecific ASIA-related manifestations eventually progressed over time to a full-blown immune disease following subsequent vaccine reexposure.^31^ Moreover, in all of our cases, several common features were observed, namely, a personal or familial susceptibility to autoimmunity and an adverse response to a prior dose of the vaccine, both of which were associated with a higher risk of postvaccination full-blown autoimmunity.^31^

### POTS and Vaccinations

**Figure 1.**
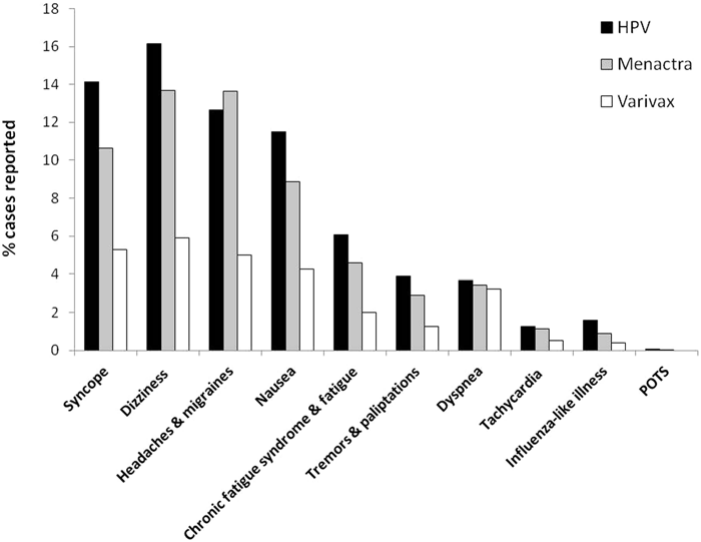
Number of adverse event reports related to POTS/CFS following HPV, Menactra meningococcal polysaccharide diphtheria toxoid conjugate, and Varivax Varicella vaccines in the US Vaccine Adverse Event Reporting System (VAERS) as of February 13, 2013. The VAERS database^33^ was searched using the following criteria: (1) Symptoms: syncope (general, exertional, postural); headaches (including migraines); nausea; chronic fatigue syndrome (including general fatigue); tremors and palpitations; dyspnea (general, exertional, at rest); tachycardia (including tachyarrhythmia, tachycardia paroxysomal, heart rate abnormal, heart rate increased, heart rate irregular); influenza-like illness (including viremia, viral infection); POTS; (2) Vaccine products: HPV, HPV2 (human papilloma virus bivalent), HPV4 (human papilloma virus types 6, 11, 16,1 8); MNQ (Meningococcal vaccine Menactra); Varcel (Varivax-Varicella virus live); (3) Gender (all genders); (4) Age (6 to 29 years; target age group for HPV, Menactra and Varivax vaccines); (5) Territory (the United States); (6) Date vaccinated (2007-2013; HPV vaccine postlicensure period).^34^ Adverse events related to a particular symptom are reported as percentages of the total number of events reported for the particular vaccine (ie, 14% syncope refers to the 2354 reports of syncope out of a total of 16 644 adverse events associated with the HPV vaccine; the total number of adverse events reported for Varivax and Menactra was 9136 and 8790, respectively).

Ours is the seventh case of POTS associated with the qHPV vaccine Gardasil reported in the literature. In addition, POTS following administration of the novel H1N1 influenza vaccine was reported recently.^13^ Recently, Blitshteyn^12^ reported six cases of POTS following HPV vaccination. In this case series, all six previously healthy young women (aged 12 to 22 years) developed symptoms of POTS within 6 days to 2 months after vaccination with the Gardasil HPV vaccine. Of further relevance to our case, two out of six cases reported by Blitshteyn also showed a positive ANA and, in all six cases the symptoms were disabling. In particular, three of the patients were not capable of attending school full time and one of them became wheel-chair bound like the patient described in our report. The course of POTS following HPV vaccination was similar in all six patients, with all of them improving in 2 to 3 years’ time frame with the use of standard pharmacotherapy for POTS. It is possible as emphasized by Blitshteyn^12^ that some patients with POTS are simply undiagnosed or misdiagnosed with anxiety and panic-related disorders, which leads to underreporting and a paucity of data on the incidence of POTS and other autonomic system disorders following vaccination. The analysis of the US VAERS database substantiates this concern. In particular, although the majority of POTS-related symptoms were reported in 4% to 16% of HPV vaccine recipients, POTS was only reported in 0.07% of cases (Figure 1). The highest number of both POTS- and CFS-related symptom reports was associated with HPV vaccines when compared with 2 other vaccines (Menactra and Varivax), routinely given to adolescents in the United States. On average, the number of VAERS reports related to POTS/CFS symptoms was 3 to 5 times greater for the HPV compared with the Varivax vaccine. A relatively high percentage of POTS/CFS symptom reports was also associated with the Menactra vaccine. If these symptoms were psychogenic and not related to a specific vaccine but rather a reaction to the injection procedure itself, one would expect a more even distribution of reports with different vaccines. In particular, the percentage of POTS/CFS reports for Varivax should be more or less the same as for Menactra especially considering the fact that the total number of VAERS reports associated with these 2 vaccines was roughly the same (9136 and 8790, respectively). As shown in Figure 1, this is not the case. Consistent with our findings, Slade et al^32^ found a disproportional reporting of syncope following HPV compared with other vaccines in their 2009 postlicensure analysis of adverse events reported to VAERS and published in JAMA. We are in further agreement with Slade et al^32^ who also noted that although VAERS shares inherent limitations of all passive surveillance systems, it is national in scope and can thus provide important signals that may require further attention. Indeed, because both POTS and CFS are frequently severely disabling,^1,6,9,10,13,15^ a more thorough follow-up of patients who present with relevant complaints postvaccination seems warranted in order to determine the true incidence of these syndromes with particular vaccines.

Another possible reason for the frequent misdiagnosis of POTS is that patients with this syndrome typically present with complaints that partially overlap with those seen in panic disorders or chronic anxiety.^2^ Notably, such symptoms (syncope, hyperventilation, limb jerking, numbness or tingling, palpitations, and tremors) appear to be among the most frequently reported adverse reactions following vaccination with HPV vaccines and may be mistakenly labeled as “psychogenic events.”^35^ From our patient’s medical history, it is evident however that the post-qHPV vaccination phenomena were neither temporary nor psychogenic. Indeed, the psychiatrist’s evaluation specifically excluded the latter in addition of finding no relevant signs of anxiety or panic disorders. The highly positive ANA further excludes a psychosomatic origin of the patient’s illness; rather, it suggest an immune-/auto-immune-mediated underlying pathology.

Although in our case the patient had a previous history of relevant symptoms (headaches, dizziness, photophobia, and phonophobia) 2 years prior to qHPV vaccination, all of these symptoms resolved in the same year and did not cause long-term disability of the patient. Indeed, at the time of first vaccination the patient was in good general health. Moreover, during the course of her illness, the patient experienced a wide spectrum of new-onset adverse conditions, including recurrent episodes of syncope with complete loss of consciousness, disabling fatigue, neck pain, joint pains, numbness in the legs, cognitive disturbances, blurred vision, unrefreshing sleep, tachycardia, dyspnea, impaired thermoregulation, cold extremities, blush discoloration of toes, excessive hair loss, GI disturbances, diminished appetite, altered sense of taste, and significant weight loss. She also tested positive for ANA, lupus anticoagulant, and antiphospholipid and was subsequently diagnosed with undifferentiated connective tissue disease/Raynaud’s. Notably, none of these manifestations were present prior to the onset of her illness in February 2009 following Gardasil vaccination, indicating that the vaccine may have been the triggering, or at the very least, the exacerbating factor.

Although a viral illness cannot be completely excluded as the primary trigger of POTS/CFS in our case, it should be noted that symptoms mimicking viral illness (commonly referred to as flu-like symptoms) are in fact one of the well-recognized symptom categories in CFS.^10,36^ Moreover, both flu-like symptoms and CFS are associated with the use of certain vaccines, and more specifically, aluminum and other vaccine adjuvants.^14,15,37^ Indeed, because vaccines induce an immune response similarly to infections, they may also just like infections trigger autoimmune diseases.^38^ However, unlike infectious agents, vaccines frequently contain adjuvants that further enhance their immune stimulation, above the levels of natural infections.^39^ These observations suggest that vaccines may provoke more exaggerated, anarchic immune responses than infections. The latter point is specially relevant in view of the fact that vaccines (including HPV) are typically repeatedly administered over relatively short periods of times (ie, weeks or months). Moreover, vaccines have been reported to precede CFS mainly following exposure to multiple vaccinations and/or as an adverse response to the vaccine adjuvant.^14,15,39,40^

### POTS, CFS, and the ASIA Syndrome

It is of further relevance to note that the safety trials for Gardasil (which is an aluminum-adjuvanted vaccine) did not include a true inactive placebo but rather an aluminum-adjuvant-containing placebo,^41^ despite much data showing that aluminum in vaccine-relevant exposures can be toxic to humans.^42,43^ In the last decade, studies on animal models have repeatedly demonstrated the ability of aluminum adjuvants to inflict immune-mediated diseases by themselves.^44,45^ This research culminated in delineation of ASIA (autoimmune/inflammatory syndrome induced by adjuvants), which encompasses several medical conditions with similar set of signs and symptoms and a common exposure to an immune adjuvant.^10,29^ Shoenfeld and colleagues proposed 4 major and 4 minor criteria for ASIA (Table 2), and in order to diagnose ASIA, fulfillment of either 2 major or 1 major and 2 minor criteria is required.^29^ The criteria for ASIA enable the inclusion of patients with well-defined autoimmune diseases (ie, multiple sclerosis, lupus) as well as those with ill-defined and nonspecific yet clinically relevant conditions (ie, myalgia, chronic fatigue, and cognitive disturbances) under the spectrum of vaccine adjuvant-associated conditions.^30^ The inclusion of the latter category of manifestations under ASIA is of special importance as these nonspecific manifestations are all too easily ignored or disregarded as irrelevant and nonvaccine related not only by patients and physicians but also by scientists involved in design of vaccine trials.^46,47^ Nonetheless, many ill-defined medical conditions that fall under the ASIA spectrum are frequently disabling and thus of significant clinical relevance. For example, CFS and cognitive dysfunction associated with the aluminum vaccine adjuvant-induced macrophagic myofasciitis (MMF) syndrome are disabling in 87% and 53% of cases, respectively,^9^ and impair both professional activities as well as numerous aspects of daily life.^9,42^ Similarly in our case, the patient was unable to attend regular school due to progressive and disabling POTS/CFS symptoms. In addition, some of the nonspecific ASIA manifestations have the potential to progress over time to a full-blown autoimmune disease, especially following subsequent vaccine re-exposure.^31^ Of note, our patient fulfilled the first 2 major criteria for ASIA (due to a prior exposure to the HPV vaccine and the obvious appearance of “typical” manifestations) as well as 3 minor criteria, owing to the positive ANA, lupus anticoagulant, and antiphospholipid and the concurrent diagnosis of Raynaud’s (Table 2).

In years following licensure, numerous case reports of serious adverse reactions of the autoimmune origin associated with the qHPV vaccine Gardasil have raised concerns about the safety of the vaccine.^12,31,48-52^ Postlicensure data from vaccine safety surveillance databases worldwide appear to substantiate these concerns. For example, in the United States, compared with all other vaccines Gardasil alone is associated with >60% of all serious adverse reactions (including 63.8% of all deaths and 81.2% cases of permanent disability) in females younger than 30 years of age.^34^ These observations suggest that HPV vaccine risks may not have been fully identified during prelicensure trials.^34,41,53^ The unusual frequency of adverse reactions following HPV vaccination cannot solely attributed to the aluminum adjuvant, as many other vaccines also contain aluminum (ie, tetanus, diphtheria, etc) but are not associated with as many adverse reactions. However, it is the aluminum that evokes the enhanced immune reaction necessary for inducing the production of the elevated titers of antibodies. The antigen on its own is not capable of evoking this strong immune response. Because of this, any adverse effect arising from the antigen (or other constituents in the vaccine) is ultimately linked to the action of the adjuvant. For example, Zivkovic et al^54^ showed that induction of the antiphospholipid syndrome (APS) syndrome and associated decreased fecundity by tetanus toxoid (TTd) hyperimmunization in C57BL/6 mice critically depends on the aluminum adjuvant. In particular, Zivkovic et al^54^ investigated reproductive pathology induced in C57BL/6 mice by TTd hyperimmunization using a combination of different pretreatments (complete Freund’s adjuvant or glycerol) and adjuvants (aluminum-hydrogel or glycerol). A decrease in fecundity was recorded in only C57BL/6 mice immunized with aluminum-hydrogel adjuvant, irrespective of the kind of applied pretreatment.

In conclusion, herein we described a case of disabling CFS/POTS secondary to qHPV Gardasil vaccination with symptom onset at 2 months following the second vaccine booster. With the concurrent detection of elevated ANA, lupus anticoagulant, antiphospholipid, and subsequent diagnosis of Raynaud’s, this case fully meets the criteria for the recently identified ASIA syndrome (Table 2). Moreover, the case presented here is consistent with other literature supporting an immune-mediated etiology of POTS and CFS.^1,12,13,15-17^ To the best of our knowledge, this is the second case of post-HPV vaccine associated POTS described in the literature to date. Due to the wide heterogeneity of symptoms and its frequent co-occurrence with other systemic autoimmune diseases, POTS is difficult to diagnose and hence many cases remain unreported. The relatively high prevalence of POTS/CFS-related symptoms in young women vaccinated with HPV vaccines (Figure 1) should alert physicians to a closer monitoring of post-HPV-related manifestations fitting the POTS/CFS criteria. We also recommend further studies to ascertain whether or not the association between HPV vaccination and POTS is causal.
